# Bust of Prof. Mussy

**Published:** 1858

**Authors:** 


### Bust of Prof. R. D. Mussy of Ohio.

The names of good and great men should be preserved in memory, to be handed down, with their history, to future generations, that they may imitate their lives and emulate their virtues. It is true that, so long as those who live cotemporaneously and have from observation learned to admire and esteem the worth and merits of illustrious of their time, there is less necessity for memorials to awaken the recollection of that which is so vivid in the mind. And, although history in the future will record their acts, and bear evidence of their usefulness during their life career, yet as this recorded proof of their superiority will only meet the eye of the comparatively few, it is meet that there should be substantial objects at every turn to awaken memory anew, and start enquiry into the history of those whose race is run, and now repose from their labors and cares in the silent tomb.

Such tokens, erected by a grateful people, who live in immediate relation with the subjects of the tribute, serve as suggestive objects to those who succeed them into the object and purpose of their erection, hence the story of their lives is rehearsed again and again to those who come after the period which closed the career of the honored dead.

With this view, copies in plaster of the bust of the deservedly distinguished veteran and father in medicine in the west, whose name is at the head of this article, and because we venerate the man and honor him for his learning and skill as a surgeon, physician and instructor. We would be gratified with the possession of one of the copies, and there are others, doubtless, who once knew him would also be pleased to possess such a remembrances here in the North-west.

Medical men should, in this manner, honor the names and character of the prominent men of their own honored calling. If we take a seat in the cabinet of a statesman, we will find there the likenesses of other conspicuous men of his class. If we wander into the library of the divine, we will have our attention arrested by the engraved resemblances of the reverend and pious advocates and followers of their Divine Master, who have lived a life of stern piety, and whose zeal and devotion have made them conspicuous and prominent in the clerical record. And in the home of the valorous soldier and military chief, the walls are made almost a dress parade with the number and high rank of the patriots and heroes. In the studio of the literary devotee, also, there is seen enough to revive the recollection of those who have largely and laboriously contributed to the literature of the world.

Medical men, in like manner, should give evidence of their appreciation of the important services of those who have proved benefactors of their species, by the cultivation of that science which is inferior to none for the practical good which flows from its exercise among mankind.

Although the original is in the sear and yellow leaf of life, with his declining sun silvering his locks, we trust he may yet be spared among us, to inculcate by precept, the benefits of a life long experience, and by his example, to dignify a profession which he has so much adorned.

We trust other cities may follow the example of Cincinnati in this respect, and give to the profession substantial memorials of other hoary veterans who yet stand as prominent and shining marks in the profession of our country.

